# Measurement of Salivary Cortisol for Revealing Age-Specific Dependence of Cortisol Levels on Time, Feeding, and Oxygen Metabolism in Newborn Infants

**DOI:** 10.3390/bios15070420

**Published:** 2025-07-01

**Authors:** Tomoko Suzuki, Sachiko Iwata, Chinami Hanai, Satoko Fukaya, Yuka Watanabe, Shigeharu Nakane, Hisayoshi Okamura, Shinji Saitoh, Osuke Iwata

**Affiliations:** 1 Center for Human Development and Family Science, Department of Pediatrics and Neonatology, Nagoya City University Graduate School of Medical Sciences, Nagoya 467-8601, Japan; 2 Centre for Developmental and Cognitive Neuroscience, Kurume University School of Medicine, Kurume 830-0011, Japan

**Keywords:** adrenal function, age, cortisol, hypothalamus–pituitary–adrenal axis, interaction, newborn infant, nutrition, oral feeding, preterm birth, stress, pain

## Abstract

Salivary cortisol is widely used to assess stress and circadian rhythms, yet its control variables in neonates, particularly regarding postnatal age, remain poorly understood. To elucidate age-specific effects of clinical variables on cortisol levels, 91 neonates with a mean (standard deviation) gestational age of 34.2 (3.8) weeks and postnatal age of 38.3 (35.4) days were categorised into Early, Medium, and Late groups by quartiles (days 10 and 56). Interactions with postnatal age were evaluated by comparing Early-to-Medium or Early-to-Late differences in regression coefficients between independent variables and cortisol levels. In the whole cohort, maternal hypertensive disorders of pregnancy and morning sampling were associated with reduced cortisol levels (both *p* = 0.001). Mean regression coefficients (95% CI) between variables and cortisol levels were as follows: for postconceptional age, Early, −0.102 (−0.215, 0.010) and Late, 0.065 (−0.203, 0.332) (*p* = 0.035); for feeding duration, Early, 0.796 (−0.134, 1.727) and Late, −0.702 (−2.778, 1.376) (*p* = 0.010); for time elapsed since feeding, Early, −0.748 (−1.275, −0.221) and Late, −0.071 (−1.230, 1.088) (*p* = 0.036); and for blood lactate, Early, 0.086 (0.048 to 0.124), Medium, 0.022 (−0.063, 0.108), and Late, −0.018 (−0.106, 0.070) (*p* = 0.008 and <0.001 vs. Medium and Late, respectively). The influence of postconceptional age, oral feeding, and anaerobic metabolism on salivary cortisol levels was observed during the birth transition period but not beyond 10 days of life. Given the age-specific dependence of cortisol levels on clinical variables, including postconceptional age, feeding, and oxygen metabolism, caution is warranted when interpreting findings from studies on salivary cortisol in newborn infants.

## 1. Introduction

Advances in neonatal care have significantly improved outcomes following preterm births. In Japan, survival rates for preterm infants born at 22 and 23 weeks of gestation increased from 31% and 56%, respectively, between 2002 and 2004, to 63% and 80%, respectively, between 2018 and 2020 [1,2]. A similar improvement was observed in the incidence of destructive cerebral injury among survivors. For instance, periventricular leucomalacia was reported in 7.9% of infants born at less than 34 weeks of gestation in a study from 1990 to 1991, which declined to 3.3% by 2007 [3]. Nevertheless, studies have indicated no corresponding reduction in the incidence of cognitive impairments following preterm birth [4,5,6]. Furthermore, accumulating evidence suggests that preterm birth is specifically associated with altered higher cognitive functioning, with markedly increased rates of attention deficit–hyperactivity disorder and autism spectrum disorder compared with term-born peers [4,7,8,9]. Collectively, preterm birth remains a substantial risk factor for a spectrum of cognitive dysfunctions.

Cerebral dysfunction in preterm infants is primarily attributed to hypoxic–ischaemic cerebral injury resulting from placental dysfunction and immature respiratory and circulatory regulation after birth. Coexisting conditions, such as inflammation and malnutrition, are known to influence the type and severity of cerebral injury. Given that the impact of preterm birth on regional brain growth and function is observed in a dose-dependent manner across extremely preterm to late preterm infants [10,11], subtle variables, such as environmental factors and sensory inputs, may contribute to higher cognitive dysfunction. The influence of noxious stimuli on the functioning of the immature brain has rigorously been explored [12,13], based on the understanding that preterm infants are more sensitive to pain than older children and adults [14]. These studies demonstrate that frequent painful procedures in preterm infants are associated with less complex cerebral microstructures and poorer cognitive development [15,16,17]. Optimised sensory inputs, aimed at reducing noxious stimuli, may therefore represent a novel strategy to ameliorate neurocognitive disorders, alongside interventions targeting hypoxia–ischaemia and other classical causes of cerebral injury [18,19,20,21,22].

To develop a novel preventive approach to improve cognitive outcomes in preterm infants through optimised sensory inputs, monitoring pain and stress in infants is essential. However, no established method exists for quantifying pain and stress in newborn infants, which complicates the development of novel strategies, especially because infants cannot verbally express their experience of noxious stimuli. Salivary cortisol has long been used as a surrogate marker of stress in adults, and this non-invasive technique has been adopted in studies of children and infants over the past 30 years. Significant increases in salivary cortisol levels have been observed in newborn infants following painful procedures [23,24,25]. However, inter-individual variability and fluctuations in cortisol levels limit its reliability as a surrogate marker of pain and stress in this population. Elevated serum cortisol levels in hospitalised newborns are associated with various clinical conditions, including younger gestational and postnatal ages, severe lung disease, use of respiratory support devices, and oral feeding [26,27]. Furthermore, infant maturation, represented by gestational age at birth, postnatal age, and postconceptional age, is known to modify the influence of independent variables on cortisol levels [28]. Therefore, before salivary cortisol can be used as a surrogate marker of stress and pain, its dependence on clinical background variables must be thoroughly elucidated, particularly in relation to differences in infant age.

This study aimed to identify independent variables influencing salivary cortisol levels and their age-specific roles in hospitalised but clinically stable preterm and term infants.

## 2. Materials and Methods

### 2.1. Ethics Approval and Consent

The protocol for this prospective observational study was approved by the Ethics Committee of Nagoya City University Graduate School of Medical Sciences, Nagoya, Japan (approval number: 60-20-0157). Written informed consent was obtained from the parents of each newborn infant. Physiological data, including salivary cortisol measurements, were collected according to the study protocol under minimal-risk conditions for the infants. To ensure privacy protection, all directly identifying information was removed and replaced with a unique study ID. A table of original IDs and corresponding pseudonymous study IDs was maintained separately in a locked, access-controlled cabinet; only de-identified data labelled by study ID were used in all analyses.

### 2.2. Study Population

This study formed part of a broader investigation aimed to establish an objective marker of pain in newborn infants. Between February 2022 and September 2024, 77 newborn infants, born at 22–41 weeks of gestation and hospitalised at the tertiary neonatal intensive care unit (NICU) of Nagoya City University Hospital, were enrolled after weaning from intensive care interventions, such as venous drip infusion and invasive or non-invasive positive pressure ventilation. Infants with major congenital anomalies, chromosomal abnormalities, severe intracranial haemorrhage, necrotising enterocolitis requiring surgical intervention, or sepsis were excluded to minimise potential bias. Some infants were enrolled multiple times at intervals of at least 2 weeks.

### 2.3. Data and Sample Collection

Data and sample collection were scheduled for a day when a small amount of blood (up to 300 μL) was required for clinical purposes. Before blood sampling, infants were fed orally at either 8:00 or 14:00. In the unit, cycled lighting was provided, targeting 100–200 lx during the daytime and 10–30 lx at night. Standard electroencephalogram cup electrodes and 16-channel near-infrared topographic spectroscopy probes were attached when the infant showed signs of sleepiness. The entire body of the infant was continuously video recorded from 40 cm above the head throughout this study. After the infants had been calm or asleep for at least 5 min, baseline saliva samples were collected using an absorbent swab stick (Saliva Bio; Salimetrics LLC, State College, PA, USA). The swab method was employed in accordance with previous studies that identified it as a suitable approach for collecting non-stimulated whole saliva in newborn infants [29,30]. No specific filter was used for saliva extraction, as the physical structure of the sponge material itself effectively removes impurities such as mucus and epithelial cells [31]. After collection, the swab stick was immediately centrifuged for 60 s in a swab basket, allowing for saliva to drain to the bottom of the tube [32]; if the saliva volume was less than 50 μL, sampling was repeated within 3 min of the first attempt. Regardless of the saliva volume obtained, the sample was centrifuged for 5 min and stored at −80 °C until assay. Following a 5 min baseline data collection, blood sampling was performed via heel lance using an automatic lancet (Quikheel Infant Incision Lancet, Becton Dickinson, Franklin Lakes, NJ, USA), typically completed within 30 s. Blood gas and lactate levels were measured using a bedside analyser (ABL 90 FLEX Plus, Radiometer, Copenhagen, Denmark). Additional saliva samples were collected at 10 and 30 min post-blood sampling; however, only samples obtained before blood sampling were analysed in this study.

### 2.4. Cortisol Assay

Cortisol levels were measured using an enzyme immunoassay kit (High-Sensitivity Salivary Cortisol Enzyme-Linked Immunosorbent Assay Kit; Salimetrics LLC, State College, PA, USA). The assay’s limit of detection was 0.19 nmol/L, with intra- and inter-assay coefficients of variation of 5.4% and 6.4%, respectively.

### 2.5. Clinical Data

During data collection, the volume of milk consumed, duration of oral feeding, and time elapsed from feeding to saliva collection were recorded. Additional clinical information was retrieved from the electronic patient records, including maternal age at delivery, gestational age, delivery mode, premature rupture of membranes, histopathologically confirmed chorioamnionitis, hypertensive disorders of pregnancy (HDP), antenatal glucocorticoid administration, sex, birth weight, Apgar scores, need for mechanical ventilation, incidence of bronchopulmonary dysplasia, requirement for postnatal glucocorticoids, and milk type on the study day. The standard score for birth weight was calculated, and intrauterine growth restriction was defined as below the 10th percentile of the norm [33]. Based on video recordings taken shortly before saliva collection, an experienced examiner (S.T.) assessed behavioural and physiological indicators—including facial expression, cry, breathing patterns, arm and leg movements, and state of arousal—to assign a composite Neonatal Infant Pain Scale (NIPS) score ranging from 0 to 7 [34].

### 2.6. Data Analysis

Values are reported as incidence (%), mean (standard deviation), or mean (95% confidence interval range), unless otherwise specified. Cortisol levels were standardised using natural logarithms. All statistical analyses were performed using SPSS software (ver. 27, IBM, Armonk, NY, USA).

The dependence of cortisol levels on clinical variables was initially evaluated using a generalised estimating equation, without considering the effect of postnatal age. Infant identity was included as a fixed variable to account for repeated sampling. Statistical significance was set at *p* < 0.003, adjusting for multiple comparisons across the 16 independent variables.

To investigate whether the dependence of salivary cortisol levels on clinical background variables changed with postnatal age, a generalised estimating equation was used to assess the dependence of cortisol levels on representative clinical variables, accounting for their interactions with postnatal age. The study population was divided into Early, Medium, and Late age groups based on the lower and upper quartiles of postnatal age. Independent variables were selected from the following domains: age and time, treatments and stressors, hypothalamus–pituitary–adrenal axis, and feeding [35]. Continuous independent variables were mean-centred for convenience. Interactions with postnatal age were assessed by Early-to-Medium or Early-to-Late differences in (i) regression coefficients between numeric variables and cortisol levels or (ii) effects of categorical variables on cortisol levels. When examining interactions between postnatal age and categorical variables, findings from contingency table cells with an incidence of less than 10% of the corresponding row or column total were excluded due to limited statistical power.

## 3. Results

A total of 77 infants were assessed for 100 times; however, saliva samples from 9 infants were insufficient for the assay. Sensitivity analysis revealed no significant differences in the clinical backgrounds between infants with sufficient and insufficient saliva samples. Infants with insufficient samples were not considered further.

Ninety-one saliva samples from 77 infants were assessed. The final study cohort was 1916 (677) g and 34.2 (3.8) weeks gestation at birth; 27 (38.6%) infants were male and 53 (75.7%) were born via Caesarean section (Table 1). Saliva samples were collected at 38.3 (35.4) days post-birth, corresponding to a postconceptional age of 38.7 (2.2) weeks. The NIPS score at the commencement of saliva collection was 0.6 (1.5). Morning samples were obtained in 72 (79.1%) studies. The cohort was divided into Early (≤10 days, *n* = 22), Medium (11–56 days, *n* = 45), and Late (>56 days, *n* = 24) age groups. Clinical characteristics of the entire cohort and the three postnatal age subgroups are presented in Appendix A. Salivary cortisol values were 20.0 (18.5) nmol/L for the entire cohort and 25.0 (26.8) nmol/L, 19.3 (16.2) nmol/L, and 16.9 (12.1) nmol/L for the Early, Medium, and Late age groups, respectively. A trend towards declining cortisol levels with increasing age was observed, though statistical significance was not reached.

### 3.1. Crude Associations Between Clinical Backgrounds and Cortisol Levels

Lower salivary cortisol levels were associated with maternal HDP and morning sample collection (both *p* = 0.001). A trend towards lower cortisol levels was noted for delivery via emergency Caesarean section, but this association was not significant after Bonferroni correction (*p* = 0.030, Table 2).

### 3.2. Influence of Postnatal Age to the Associations Between Clinical Backgrounds and Cortisol Levels

**Figure 1:**
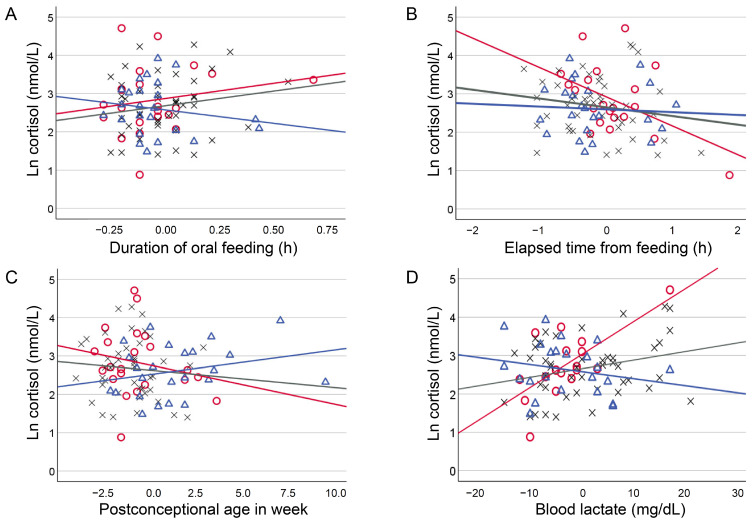
Influence of postnatal age to the relationship between numeric clinical variables and cortisol levels. Scatter plots depicting the influence of postnatal age to the relationship between mean-centred numeric clinical variables and ln cortisol values. Postnatal age was classified into three groups of Early (≤10 d, open red circles with red regression lines), Medium (10 < Medium ≤ 56 d, grey crosses with grey regression lines), and Late (56 d<, open blue triangles with blue regression lines) using the quartiles.

**Figure 2:**
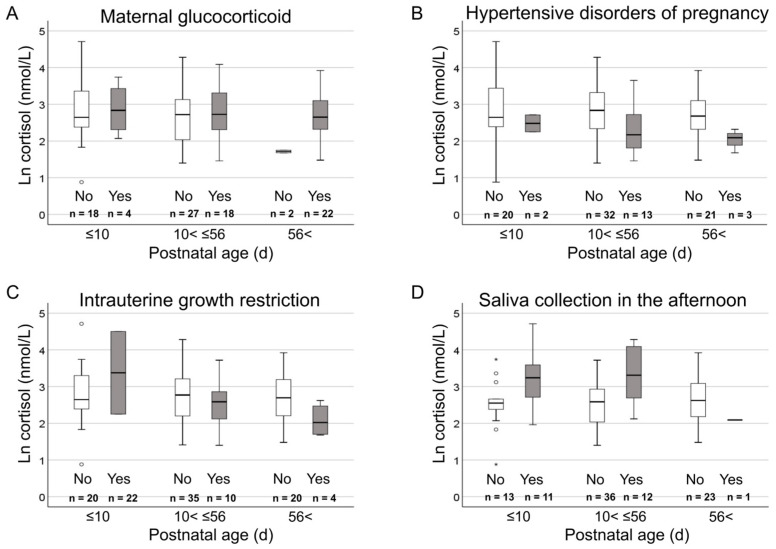
Influence of postnatal age to the relationship between categorical clinical variables and cortisol levels. Box plots depicting the relationship between categorical clinical variables (A Maternal glucocorticoid, B Hypertensive disorders of pregnancy, C Intrauterine growth restriction, and D Saliva collection in the afternoon.) and ln cortisol values for three postnatal age groups of Early (≤10 d), Medium (10 d < Medium ≤ 56 d), and Late (56 d <). Postnatal age was classified into three groups of Early (≤10 d), Medium (10 < Medium ≤ 56 d), and Late (56 d <) using the quartiles. Symbols: box, first, second, and third quartiles; perpendicular line, range without outliers; open circle, mild outliers more than 1.5 times and less than or equal to 3 times the interquartile range from the first quartile; and asterisks, extreme outliers more than 3 times beyond the interquartile range from the first quartile.

The relationships between clinical background variables and cortisol levels showed significant interactions with postnatal age in four continuous numeric variables: postconceptional age, time required for feeding, elapsed time from feeding, and blood lactate levels (Table 3 and Figure 1 and Figure 2). Regression coefficients for these variables were as follows: for postconceptional age, Early, −0.102 (−0.215, 0.010) and Late, 0.065 (−0.203, 0.332) (*p* = 0.035); for feeding duration, Early, 0.796 (−0.134, 1.727) and Late, −0.702 (−2.778, 1.376) (*p* = 0.010); for time elapsed since feeding, Early, −0.748 (−1.275, −0.221) and Late, −0.071 (−1.230, 1.088) (*p* = 0.036); and for blood lactate levels, Early, 0.086 (0.048 to 0.124); Medium, 0.022 (−0.063, 0.108); and Late, −0.018 (−0.106, 0.070) (*p* = 0.008 for Medium and *p* < 0.001 for Late).

## 4. Discussion

This study investigated the independent variables influencing salivary cortisol levels and their age-specific roles in a relatively large cohort of hospitalised newborn infants. Lower salivary cortisol levels were associated with maternal HDP and morning sample collection across the entire cohort, confirming previous findings from smaller cohorts [24,25,28]. By contrast, an age-specific dependence of salivary cortisol levels on clinical background variables was newly identified. Trends towards higher cortisol levels were observed in association with younger postconceptional age, longer oral feeding duration, shorter time elapsed since feeding, and higher blood lactate levels, but only during the first 10 days of life. Since the relationship of salivary cortisol levels on clinical variables is likely to change with age, a deeper understanding of the independent variables and their interactions with age is essential before salivary cortisol can be reliably used as a surrogate marker of pain, stress, and circadian rhythms in newborn infants.

### 4.1. Time of Day and Cortisol Levels

Previous studies on small cohorts of hospitalised newborn infants consistently reported higher salivary cortisol levels in the afternoon than in the morning [24,25]. These findings have been attributed to the persistence of the foetal adrenal rhythm, which is entrained in antiphase to maternal rhythms [36,37]. In this study, higher cortisol levels were observed in afternoon saliva samples compared with morning samples throughout the study period, confirming this pattern in a larger cohort. This suggests that the mild day/night light cycle provided in neonatal intensive care units (NICUs), including the current study setting, is insufficient to promote mature diurnal rhythms and may even be deleterious, as foetal-type rhythms persisted for weeks post-birth. The establishment of morning-dominant diurnal cortisol rhythms typically requires approximately 3 months, even in healthy newborn infants [38]. However, an observational study of 1300 infants demonstrated that a home environment facilitates the development of a nighttime-dominant sleep pattern in healthy infants within the first month of life [39]. Given that an infant’s nighttime sleep is crucial for the mother’s own sleep and mental health [40], the NICU environment may require enhancement through increased daytime stimulation. Such improvements could help ease the transition to home care and reduce difficulties after discharge from the NICU.

### 4.2. Maternal Condition, Exogenous Glucocorticoids, and Cortisol Levels

A previous study reported that antenatal maternal glucocorticoid administration attenuates plasma cortisol levels in preterm infants at 7 days but not at 14 days of life [41]. In this cohort, at least four weeks had elapsed since antenatal glucocorticoid administration for affected infants, which likely explains the lack of correlation between antenatal glucocorticoid and cortisol levels in infants. Similarly, postnatal administration of glucocorticoids was not associated with salivary cortisol levels, presumably because only 3% of the infants were studied within 14 days after the final glucocorticoid administration. Beyond exogenous glucocorticoids, maternal HDP and the birth of small-for-gestational-age infants are known to affect hypothalamus–pituitary–adrenal axis regulation [28]. In the current study, maternal HDP was associated with lower cortisol levels in infants, a trend that was unaffected by postnatal age. For small-for-gestational-age infants, trends towards higher cortisol levels in the Early age group and lower levels in the Medium age group were observed (the Late group was not assessed due to low incidence), though these trends were not statistically significant. These findings align with previous reports showing an association between small-for-gestational-age and elevated cortisol levels shortly after birth, followed by lower cortisol levels after the second week of life [28,42].

### 4.3. Age, Maturation, and Cortisol Levels

Appropriate adrenal function regulation is critical for the maturation of immature organs [35]. Mori et al. demonstrated reduced plasma cortisol levels in newborns with lower gestational age at birth [42]. In our current study, preterm infants were enrolled only after clinical stabilisation, which may explain the lack of association between gestational age at birth and salivary cortisol levels. In contrast, the relationship between postconceptional age and cortisol levels shifted from negative in the Early age group to positive in the Late age group. This supports the hypothesis that adrenal function in immature infants is initially accelerated during the birth transition but subsequently declines to levels comparable to, or below, those of more mature infants. Prospective studies enrolling infants at fixed postnatal ages are required to assess the unbiased effects of postconceptional age on cortisol levels, accounting for maternal, placental, and clinical variables, including exogenous glucocorticoid administration.

### 4.4. Feeding and Cortisol Levels

In adults, plasma cortisol levels increase postprandially [43,44]. Our previous study in hospitalised newborn infants confirmed a feeding-induced elevation in salivary cortisol levels; however, this trend was observed only following oral feeding and not gavage feeding [27]. This suggests that feeding-induced stress, rather than an enteral nutrition-induced response, may stimulate cortisol secretion from the adrenal gland. In the current study, a trend towards higher cortisol levels was observed with shorter elapsed time between feeding and saliva sampling, indicating a possible cortisol elevation after oral feeding followed by a temporal decline—though this was not statistically significant. When stratified by postnatal age, the relationship between elapsed time after feeding and cortisol levels shifted from negative in the Early age group to neutral in the Late age group. Similarly, the relationship between the duration of oral feeding and cortisol levels changed from positive in the Early age group to negative in the Late age group. These findings suggest that immature oral feeding—particularly prolonged feeding—may stimulate adrenal cortisol secretion, particularly during the transitional period after birth.

### 4.5. Blood Lactate and Cortisol Levels

Elevated blood lactate levels typically reflect increased anaerobic metabolism [45]. A positive relationship between blood lactate levels and salivary cortisol levels was observed in the Early age group but not in the Medium or Late age groups. Given that blood sampling is typically completed within 30 s after heel lancing, the measured lactate levels are likely to reflect the infants’ resting-state oxygen metabolism or responses to stimuli occurring prior to saliva sampling, rather than an acute response to the heel lance itself. Infants in the Early age group were undergoing the transition from the intrauterine environment, during which anaerobic metabolism remains relatively elevated [46]. For these vulnerable infants, even subtle stimuli—such as oral feeding—may act as stressors, leading to simultaneous elevations in blood lactate and salivary cortisol levels.

### 4.6. Strength and Limitations

Our study was conducted as part of a larger project ultimately aimed at reducing the impact of procedural pain and stress in newborn infants. However, the current study represents the starting point of the project and was conducted as a preliminary investigation to identify independent variables affecting salivary cortisol levels so that this biomarker can be used in newborn infants with minimal bias. Although the sample size was relatively larger than that of previous studies with similar scopes, the population was still too small as a cross-sectional cohort to fully elucidate the control variables of salivary cortisol levels in newborn infants. Nonetheless, we were able to confirm several important but previously uncertain independent variables influencing salivary cortisol levels, and newly demonstrated that the relationship between clinical variables and salivary cortisol levels is dependent on postnatal age. These findings may help better understand the complex but critical regulation of adrenal function in immature and ill newborn infants. It should also be noted that infants were enrolled across a wide range of maturational stages after weaning from intensive care, introducing potential bias in the clinical backgrounds of the three postnatal age groups. Infants born at younger gestational ages often required longer stabilisation periods before enrolment. In addition, greater postnatal age was associated with higher incidences of antenatal glucocorticoid administration, premature rupture of membranes, emergency Caesarean delivery, mechanical ventilation, and chronic lung disease (see Appendix A for comparisons of variables across postnatal age groups). Finally, although the role of intrauterine growth restriction in cortisol secretion was examined, the influence of postnatal nutrition and growth could not be assessed. These limitations must be considered when interpreting the findings and designing future studies.

## 5. Conclusions

In a relatively large cohort of hospitalised newborn infants, maternal HDP and morning saliva collection were confirmed as robust control variables for adrenal cortisol secretion, with salivary cortisol levels approximately half those of unaffected infants. Additionally, postconceptional age, feeding duration, time elapsed since feeding, and blood lactate levels were identified as age-dependent variables when their interactions with postnatal age were incorporated into the model. Trends towards higher salivary cortisol levels were observed in association with younger postconceptional age, longer oral feeding duration, shorter time elapsed since feeding, and higher blood lactate levels, specifically within the first 10 days of life during the birth transition period. However, these findings were inferred from cross-sectional observations; therefore, temporal changes in the relationship between clinical variables and salivary cortisol levels should be confirmed in future longitudinal studies. Such investigation is essential for ensuring accurate interpretation of findings in studies that use salivary cortisol as a surrogate marker of pain, stress, and circadian rhythms in newborn infants.

## Figures and Tables

**Table 1:** Clinical backgrounds of the study population.

Variables	Value
Antenatal variables (*n* = 77)	
Maternal glucocorticoid	28 (40.0%)
Hypertensive disorders of pregnancy	15 (21.4%)
Premature rupture of membranes	17 (24.3%)
Variables at birth (*n* = 77)	
Caesarean delivery	
All	53 (75.7%)
Emergency	44 (62.9%)
Male infants	27 (38.6%)
Gestational age (weeks)	34.2 (3.8)
Body weight (grams)	1916 (677)
Intrauterine growth restriction	14 (20.0%)
Apgar score (1 min)	7 (6, 8)
Apgar score (5 min)	8 (7, 9)
Variables at the time of study (*n* = 91)	
Postconceptional age (weeks)	38.7 (2.2)
Postnatal glucocorticoid	23 (25.3%)
Time since the final administration (days) *	28.5 (22.5)
Administration within 14 days of study	3 (3.3%)
Administration within 28 days of study	7 (7.7%)
Feeding duration (hours)	0.33 (0.17)
Time elapsed from feeding to study (hours)	1.27 (0.59)
Morning studies	72 (79.1%)
Neonatal Infant Pain Scale before saliva collection	0.6 (1.5)
Blood pH **	7.34 (0.04)
Blood lactate (mg/dL) **	25.1 (8.6)
Cortisol (nmol/L)	20.0 (18.5)
Ln cortisol (nmol/L)	2.70 (0.76)

Values are shown as incidence (%), mean (standard deviation), or median (quartile range). Data represent 91 studies conducted in 77 newborn infants. * Calculated for 22 infants with postnatal glucocorticoid administration. ** Blood gas and lactate levels were not measured in six infants.

**Table 2:** Effect of clinical variables on cortisol levels.

Variables		Ln Cortisol (nmol/L)	Regression Coefficient	*p*
Antenatal glucocorticoid	Yes	2.73 (0.64)	0.06 (−0.270, 0.369)	0.723
	No	2.67 (0.13)	Reference	
HDP	Yes	2.28 (0.56)	−0.524 (−0.839, −0.210)	**0.001**
	No	2.80 (0.77)	Reference	
PROM	Yes	2.79 (0.71)	0.137 (−0.227, 0.500)	0.461
	No	2.65 (0.79)	Reference	
Caesarean delivery				
All	Yes	2.64 (0.73)	−0.309 (−0.780, 0.162)	0.198
	No	2.92 (0.90)	Reference	
Emergency	Yes	2.57 (0.67)	−0.414 (−0.787, −0.041)	**0.030**
	No	2.97 (0.88)	Reference	
Male infants	Yes	2.66 (0.69)	−0.068 (−0.395, 0.259)	0.683
	No	2.72 (0.81)	Reference	
Gestational age (weeks)			0.015 (−0.032, 0.061)	0.535
IUGR	Yes	2.54 (0.81)	−0.189 (−0.618, 0.240)	0.389
	No	2.73 (0.75)	Reference	
Apgar score (5 min)			0.029 (−0.166, 0.224)	0.772
Postconceptional age (weeks)			−0.009 (−0.077, 0.059)	0.793
Postnatal glucocorticoid	Yes	2.74 (0.74)	0.061 (−0.341, 0.462)	0.768
	No	2.68 (0.78)		
Feeding duration (hours)			0.476 (−0.394, 1.347)	0.284
Time elapsed from feeding to study (hours)			−0.260 (−0.557, 0.037)	0.086
Morning studies	Yes	2.55 (0.67)	−0.691 (−1.108, −0.273)	**0.001**
	No	3.24 (0.86)	Reference	
Neonatal Infant Pain Scale			−0.037 (−0.165, 0.091)	0.569
Blood pH			−0.047 (−0.096, 0.003)	0.064
Blood lactate (mg/dL)			0.019 (−0.004, 0.043)	0.106
Postnatal age (days)				
Early ≤ 10		2.83 (0.83)	Reference	
10 < Medium ≤ 56		2.68 (0.75)	−0.153 (−0.574, 0.267)	0.475
56 < Late		2.61 (0.68)	−0.224 (−0.728, 0.280)	0.384

Values are presented as mean (standard deviation) or mean (95% confidence interval range). Cortisol values are transformed into natural logarithm (Ln). *p*-values less than 0.05 are shown in **bold**. Abbreviations: HDP—hypertensive disorders of pregnancy; PROM—premature rupture of membranes; IUGR—intrauterine growth restriction.

**Table 3:** Effect of postnatal age on the regression coefficient between clinical variables and cortisol levels.

Variables		Regression Coefficient	*p*
Premature rupture of membranes		0.069 (−0.594, 0.733)	0.838
Interaction with postnatal age	Early	Reference	
	Medium	0.051 (−0.853, 0.955)	0.911
	Late	0.357 (−0.536, 1.251)	0.433
Male infants		0.428 (−0.180, 1.035)	0.168
Interaction with postnatal age	Early	Reference	
	Medium	−0.598 (−1.347, 0.152)	0.118
	Late	−0.845 (−1.764, 0.073)	0.071
Gestational age (weeks)		−0.085 (−0.199, 0.029)	0.143
Interaction with postnatal age	Early	Reference	
	Medium	0.130 (−0.030, 0.290)	0.110
	Late	−0.074 (−0.299, 0.150)	0.516
Apgar score (5 min)		0.113 (−0.228, 0.455)	0.516
Interaction with postnatal age	Early	Reference	
	Medium	0.006 (−0.428, 0.440)	0.978
	Late	−0.296 (−0.736, 0.144)	0.188
Postconceptional age (weeks)		−0.102 (−0.215, 0.01)	0.074
Interaction with postnatal age	Early	Reference	
	Medium	0.057 (−0.125, 0.239)	0.54
	Late	0.167 (0.012, 0.322))	**0.035**
Feeding duration (hours)		0.796 (−0.134, 1.727)	0.093
Interaction with postnatal age	Early	Reference	
	Medium	0.239 (−1.720, 2.198)	0.811
	Late	−1.498 (−2.644, −0.351)	**0.010**
Time elapsed from feeding to study (hours)		−0.748 (−1.275, −0.221)	0.005
Interaction with postnatal age	Early	Reference	
	Medium	0.524 (−0.139, 1.187)	0.122
	Late	0.677 (0.045, 1.309)	**0.036**
Blood pH		−4.719 (−10.933, 1.496)	0.137
Interaction with postnatal age	Early	Reference	
	Medium	4.800 (−3.839, 13.439)	0.276
	Late	−5.606 (−12.854, 1.641)	0.130
Blood lactate (mg/dL)		0.086 (0.048, 0.124)	<0.001
Interaction with postnatal age	Early	Reference	
	Medium	−0.064 (−0.111, −0.016)	**0.008**
	Late	−0.104 (−0.154, −0.054)	**<0.001**

Values are presented as mean (95% confidence interval). *p*-values less than 0.05 are shown in **bold**.

## Data Availability

Data presented in this study are available upon request from the corresponding authors.

## Abbreviations

The following abbreviations are used in this manuscript:

HDP	Hypertensive Disorders of Pregnancy
IUGR	Intrauterine Growth Restriction
NICU	Neonatal Intensive Care Unit
NIPS	Neonatal Infant Pain Scale
PROM	Premature Rupture of Membranes

## Appendix A

**Table A1:** Clinical backgrounds of the postnatal age groups.

Variables	Early (*n* = 22)	Medium (*n* = 45)	Late (*n* = 24)
Antenatal variables			
Maternal glucocorticoid	4 (18.2%)	18 (40.0%)	22 (91.7%) *
HDP	2 (9.1%)	13 (28.9%)	3 (12.5%) *
PROM	5 (22.7%)	8 (17.8%)	15 (62.5%)
Variables at birth			
Caesarean delivery			
All	12 (54.5%)	38 (84.4%)	24 (100.0%) *
Emergency	5 (22.7%)	33 (73.3%)	24 (100.0%) *
Male infants	8 (36.4%)	23 (51.1%)	6 (25.0%)
Gestational age (weeks)	37.4 ± 1.9	34.2 ± 2.3	27.6 ± 1.5
Birth weight (g)	2504 ± 419	1859 ± 448	978 ± 294
IUGR	2 (9.1%) *	10 (22.2%)	4 (16.7%)
Apgar score (1 min)	8 (8, 8)	7 (7, 8)	4 (3, 6)
Apgar score (5 min)	9 (8, 9)	8 (8, 9)	7 (6, 8)
Variables at the time of study			
Postconceptional age (weeks)	38.3 ± 1.8	37.9 ± 1.5	40.4 ± 2.7
Feeding duration (hours)	0.32 ± 0.21	0.34 ± 0.15	0.32 ± 0.17
Time elapsed from feeding to study (hours)	1.46 ± 0.57	1.23 ± 0.59	1.19 ± 0.59
Morning studies	13 (59.1%)	36 (80.0%)	23 (95.8%) *
Blood pH **	7.36 ± 0.03	7.34 ± 0.05	7.33 ± 0.04
Blood lactate (mg/dL) **	22.8 ± 6.7	26.9 ± 9.4	23.0 ± 7.8

Values are shown as incidence (%), mean ± standard deviation, or median (quartile range). * Contingency table containing cells with an incidence of less than 10% of the sum of the row or column. ** Blood gas and lactate levels were not measured in six infants. Abbreviations: HDP—hypertensive disorders of pregnancy; PROM—premature rupture of membranes; IUGR—intrauterine growth restriction.
